# Blunted angiogenesis and hypertrophy are associated with increased fatigue resistance and unchanged aerobic capacity in old overloaded mouse muscle

**DOI:** 10.1007/s11357-016-9894-1

**Published:** 2016-03-12

**Authors:** Sam B. Ballak, Tinelies Busé-Pot, Peter J. Harding, Moi H. Yap, Louise Deldicque, Arnold de Haan, Richard T. Jaspers, Hans Degens

**Affiliations:** 1 School of Healthcare Science, Manchester Metropolitan University, Chester Street, John Dalton Building, Manchester, M1 5GD UK; 2 Laboratory for Myology, Move Research Institute Amsterdam, Faculty of Behavioural and Movement Sciences, Vrije Universiteit Amsterdam, Amsterdam, The Netherlands; 3 Exercise Physiology Research Group, Department of Kinesiology, FaBeR, KU Leuven, Leuven, Belgium; 4 Lithuanian Sports University, Kaunas, Lithuania

**Keywords:** Muscle aging, Hypertrophy, Angiogenesis, Aerobic capacity, Resveratrol

## Abstract

We hypothesize that the attenuated hypertrophic response in old mouse muscle is (1) partly due to a reduced capillarization and angiogenesis, which is (2) accompanied by a reduced oxidative capacity and fatigue resistance in old control and overloaded muscles, that (3) can be rescued by the antioxidant resveratrol. To investigate this, the hypertrophic response, capillarization, oxidative capacity, and fatigue resistance of *m. plantaris* were compared in 9- and 25-month-old non-treated and 25-month-old resveratrol-treated mice. Overload increased the local capillary-to-fiber ratio less in old (15 %) than in adult (59 %) muscle (*P* < 0.05). Although muscles of old mice had a higher succinate dehydrogenase (SDH) activity (*P* < 0.05) and a slower fiber type profile (*P* < 0.05), the isometric fatigue resistance was similar in 9- and 25-month-old mice. In both age groups, the fatigue resistance was increased to the same extent after overload (*P* < 0.01), without a significant change in SDH activity, but an increased capillary density (*P* < 0.05). Attenuated angiogenesis during overload may contribute to the attenuated hypertrophic response in old age. Neither was rescued by resveratrol supplementation. Changes in fatigue resistance with overload and aging were dissociated from changes in SDH activity, but paralleled those in capillarization. This suggests that capillarization plays a more important role in fatigue resistance than oxidative capacity.

## Introduction

Aging is accompanied by a progressive decline in muscle mass (sarcopenia) and power (Roseberg 1989). These changes not only result in a decreased peak performance, but also in a reduced maximal sustainable power of the muscles as a larger proportion of the available muscle mass has to be recruited for a given task. An age-related reduction in sustainable power can be aggravated by a reduced aerobic capacity to generate ATP. Together, these changes will ultimately limit the ability to perform daily life activities and reduce the quality of life (Maden-Wilkinson et al. 2015; Paterson et al. 2007).

During aging, maximal whole-body oxygen consumption per lean mass (VO_2max_) decreases (Fleg and Lakatta 1988). In rats, also aerobic power of skeletal muscle is reduced, even when convective oxygen delivery was matched in young and old muscles (Hepple et al. 2003). Such a situation can occur due to a lower oxidative enzyme activity and/or a diminished muscle capillarization, which both have been observed in aged humans (Coggan et al. 1992; Degens 1998; Hepple et al. 1997; Konopka et al. 2014) and rats (Degens et al. 1993b; Skorjanc et al. 2001). Muscle fatigue resistance during intermittent isometric contractions is related to the oxidative capacity of single muscle fibers and motor units (Degens and Veerkamp 1994), and as aerobic metabolism requires an adequate supply of oxygen via the capillaries, it is not surprising that fatigue resistance is also positively related to muscle capillarization (Hudlicka et al. 1977). It has been found that the age-related reduction in oxidative capacity may exceed the loss of capillaries, resulting in a “superfluous” capillary supply in muscles from late-middle aged rats (Hepple and Vogell 2004). In humans, the age-related rate of decline in VO_2max_ accelerates with increasing age (Fleg et al. 2005) and while superfluous capillarization may be a late hallmark of skeletal muscle aging, it is not known whether (1) this also is the case during early stages of sarcopenia, nor (2) how early age-related changes in aerobic capacity and capillarization affect muscle fatigue resistance.

Several studies indicate that the capillary supply to a muscle fiber is positively related to its cross-sectional area in both normal (Ahmed et al. 1997; Wust et al. 2009) and hypertrophied muscle (Degens et al. 1994b). This relationship is tightly regulated, as reflected by the similar time course of fiber hypertrophy and capillary proliferation in a rat model of compensatory hypertrophy (Egginton et al. 2011; Plyley et al. 1998). We therefore hypothesize that a lower muscle capillarization in old age, which would increase the diffusion distance for oxygen and thereby potentially limit muscle oxygenation (Degens et al. 1993b; van der Laarse et al. 2005), contributes to the impaired hypertrophic response observed in muscles from old rodents (Ballak et al. 2015; Degens and Alway 2003) and humans (Kosek et al. 2006; Welle et al. 1996).

Muscle fiber size and oxidative metabolism are inversely related (van der Laarse et al. 1998; van Wessel et al. 2010), which suggests that muscle fiber size and oxidative metabolism are under coordinated control. Based on this relationship, it is to be expected that the aerobic capacity decreases during muscle (fiber) hypertrophy, and even more so in the older muscles that potentially already have a less dense capillary network to start with. This has, however, not yet been investigated systematically.

If a reduced angiogenic response and impaired mitochondrial function contribute to muscle dysfunction and an attenuated hypertrophic response in old age, then agents that improve angiogenesis and mitochondrial function may normalize muscle function and the hypertrophic response in old age. Resveratrol (3,5,4′-trihydroxystilbene), a polyphenol with anti-inflammatory and antioxidant properties (Jackson et al. 2011; Ryan et al. 2010), may be such an agent. Resveratrol has been shown to enhance expression of PGC-1α, improve mitochondrial function in mouse muscle (Lagouge et al. 2006), and to increase expression of vascular endothelial growth factor (VEGF) and its receptor Flk-1 (Fukuda et al. 2006), even in old rodents (Leick et al. 2009; Suzuki et al. 1997).

The objectives of this study were to assess (1) whether the blunted muscle fiber hypertrophy is associated with attenuated angiogenesis, (2) how muscle fatigue resistance changes with age and overload and how this is related to changes in mitochondrial content and/or capillarization, and (3) whether resveratrol improves oxidative capacity and capillarization in 25-month-old mice and rescues the hypertrophic response. To investigate the effects of aging on the hypertrophic response, capillarization, aerobic capacity, and fatigue resistance in *m. plantaris*, we used 9- and 25-month-old nontreated and 25-month-old resveratrol-treated mice.

## Methods

### Ethical approval

All experiments were approved by the local animal use and care committee of the VU University Amsterdam and conformed to the Dutch Research Council’s guide for care and use of laboratory animals.

### Animals

At the terminal experiment, adult (*n* = 11) and old (*n* = 10) male C57BL/6J mice (Janvier, France) were 9 and 25 months old, respectively. Mice were kept under specific pathogen-free conditions and housed individually at 20–22 °C at a 12-h light/dark cycle. Animals were given free access to water and chow (Ssniff® S8189-S095, the same as provided at the supplier). At the age of 7.5 or 23.5 months, the *m. gastrocnemius* and *m. soleus* of the left leg were denervated to overload the *m. plantaris* for 6 weeks (Ballak et al. 2015; Degens and Alway 2003). The right leg served as internal control. Another group of old mice (old-res, *n* = 11) received chow containing 0.4 % resveratrol (98.6 % pure, *Polygonum cuspidatum* extract; 21st Century Alternative, UK) during the last 7 weeks prior to the terminal experiment, starting 1 week before induction of overload. The old mice were randomly assigned to the old control or old-res group, and all experiments were performed in a random order.

### Resveratrol administration

Since we were only interested to test whether resveratrol could reverse the age-associated blunted hypertrophic response and increase oxidative capacity, we did not include a group of young mice with resveratrol supplementation. The daily intake of resveratrol was approximately 0.4 mg per gram body mass per day, based on a food intake of about 3 g per day. The food intake was most likely similar in old and old-res mice, since body mass did not change throughout the last 6 weeks prior to the terminal experiment in neither the old nor the old-res mice (Ballak et al. 2015).

### Preparation for in situ muscle function

Fifteen minutes prior to surgery, mice received a subcutaneous injection of 0.06 mL 1 % Temgesic (Reckitt Benckiser, UK) as an analgesic and were anesthetized with 4 % isoflurane, 0.1 L min^−1^ O_2_ and 0.2 L min^−1^ air. After nociceptive responses had ceased, the level of anesthesia was maintained with 1.5–2.5 % isoflurane. A humidifier moistened the inhaled air to prevent dehydration due to respiration. The mice were placed on a heated pad to maintain body temperature at ~36.5 °C.

All experiments were performed as described previously (Ballak et al. 2014a; Degens and Alway 2003). The *m. plantaris* was dissected free from surrounding tissue while maintaining its innervation and blood supply. The proximal end of the sciatic nerve was placed over an electrode to stimulate the muscle. The distal tendon of the *m. plantaris* was dissected free and tightened with a Kevlar thread via a small steel bar to a force transducer (de Haan et al. 1989). The femur was fixed by a clamp on the condyle of the femur. During the experiment, the muscle and its surrounding were kept moist at physiological temperature (34–36 °C) with a water-saturated airflow.

### Experimental setup and fatigue measurements

Contractile properties were determined of both the overloaded and contralateral control muscle as described previously (Ballak et al. 2014a). The order of the experiments was randomized. Contractions were elicited by supramaximal electrical stimulation of the sciatic nerve at a constant current (2 mA; 200-μs pulse width). Optimal length (λ_o_) was defined as the muscle length where maximal tetanic isometric force was generated. To set λ_o_, the length at which the muscle produced maximal twitch force was determined. To fine adjust λ_o_, tetani (stimulation frequency 150 Hz, 150 ms) were applied once every 2 min. We adjusted the stimulation frequency for the fatigue test so that the force was ~40 % maximal isometric force (test modified for mice from Degens and Alway (2003)) and stimulated the muscle with 330-ms stimulation trains once every 2 s for 4 min at this predetermined frequency. Potentiation was calculated as *F*_max_/*F*_1_·100, where *F*_max_ is the maximal force during the protocol and *F*_1_ is the tetanic force during the first contraction. The fatigue index (FI) was calculated as the *F*_120_/_1_. To correct for potentiation, we also calculated the *F*_120/max_, where *F*_120_ is the force of the last contraction.

### Histology

The *m. plantaris* was embedded at λ_o_ in a gelatin-tyrode solution and frozen in liquid nitrogen (Ballak et al. 2014a). Within a month after the contraction protocol, serial 10-μm cross sections were cut from the mid-belly of the *m. plantaris* in a cryostat at −20 °C. Sections were mounted on glass slides (Menzel-Gläser, Superfrost® Plus, GER), air-dried and stored at −80 °C until further use. All chemicals were obtained from Sigma-Aldrich (The Netherlands) unless stated otherwise.

One section was stained for succinate dehydrogenase (SDH), a marker of oxidative capacity, as described previously (van der Laarse et al. 1989). SDH activity was calculated as the absorbance at 660 nm μm^−1^ section thickness per second of staining time (ΔA660 μm^−1^ s^−1^). The integrated SDH was calculated as the product of muscle fiber CSA and SDH activity.

Capillaries were stained with biotinylated lectin (*Griffonia simplicifolia*). Sections were fixed in ice-cold acetone for 15 min and washed with HEPES buffer for 5 min. After blocking with 0.1 % bovine serum albumin (BSA) in HEPES for 60 min and being washed with HEPES, the sections were incubated with hydrogen peroxide for 30 min. After washing in HEPES, the sections were incubated with lectin for an hour (Vector Laboratories, UK), washed three times with HEPES, and incubated with VIB substrate (Vector Laboratories, UK). Then, the sections were washed with dH_2_O and enclosed with glycerin-gelatin.

Serial sections were immunohistochemically stained for type I, IIA, IIX, and IIB MHC to distinguish muscle fiber types (Ballak et al. 2014a). Thereto, monoclonal antibodies specific against types I, IIA, IIX, and IIB were used; BAD5, SC-71, 6H1, and BF-F3 (Developmental Studies Hybridoma Bank, USA), respectively. In short, sections were fixated with acetone for 10 min at 4 °C and washed three times in phosphate-buffered saline (PBS) plus Tween (PBST). After blocking with 10 % normal swine serum for 30 min, sections were incubated with the primary antibody. Subsequently, sections were washed in PBST three times and incubated in the dark with secondary antibody (Alexa 488 anti-mouse, Molecular Probes) for 30 min. After washing with PBST, incubating with wheat germ agglutinin (WGA) for 20 min, washing with PBST and subsequently washing once more with PBS—all in the dark—sections were enclosed in Vectashield®-Hard Set Mounting Medium with DAPI (Vector Laboratories, USA).

### Morphometric analysis

The morphometric analysis of the muscle samples was done single-blinded, where the experimenter did not know the origin of the analyzed section. The method of capillary domains has been used to analyze muscle capillarization, fiber type composition, and the relationship between capillarization and SDH activity (Degens et al. 1992; Hoofd et al. 1985; Wust et al. 2009). Recently, a semiautomatic domain method has been developed and validated (Ballak et al. 2016), based on the calculations in AnaTis (BaLoH Software, www.baloh.nl). The capillary domain is a good representation of the oxygen supply area of a capillary (Al-Shammari et al. 2014). Briefly, coordinates of fiber outlines and capillaries were traced and stored. Capillary domains were constructed by applying Voronoi tessellation, which creates equidistant boundaries around each capillary. The fiber cross-sectional area (FCSA) and capillary domain sizes were calculated. Besides the usual indices of capillary density (CD; cap mm^−2^) and capillary-to-fiber ratio (C/F), the method allows the calculation of the capillary supply to individual fibers, even those lacking direct capillary contacts. The capillary supply to a fiber was expressed as the local capillary-to-fiber ratio (LCFR; sum of the fractions of the capillary domains overlapping a given fiber) and the capillary fiber density (CFD; LCFR divided by the FCSA of a particular fiber). The logarithmic standard deviation of the radius of the capillary domains provides an index of the heterogeneity of capillary spacing (Log_rSD_). This analysis was performed for the glycolytic and oxidative regions of the muscle separately.

### Western blot

Frozen muscle tissue (~5–10 mg; 1:40, *w*/*v*) was homogenized as described before (Ballak et al. 2015). Homogenates were centrifuged at 10,000 *g* for 10 min at 4 °C. The supernatant was immediately stored at −80 °C and the protein concentration was measured using the DC protein assay kid (Bio-Rad Laboratories, Nazareth, Belgium). Forty micrograms of protein was separated by SDS-PAGE (10–12 % gels) and transferred to PVDF membranes. Membranes were blocked with 5 % nonfat milk for 1 h and then incubated overnight (4 °C) with the following antibodies: SDH (1:10,000, Santa Cruz, UK), cytochrome c oxidase subunit 4 (COX-4) (1:1000, Abcam, Cambridge, UK), Flk-1 (1:500, Santa Cruz, UK), and eukaryotic elongation factor 2 (eEF2) (1:1000, Cell Signaling Technology, Leiden, The Netherlands). Horseradish peroxidase-conjugated anti-mouse (1:10,000) or anti-rabbit (1:5000) secondary antibodies (Sigma-Aldrich, Bornem, Belgium) were used for chemiluminescent detection. Membranes were scanned and quantified with Genetools and Genesnap software (Syngene, Cambridge, UK), respectively. Preliminary experiments showed that eEF2 content was stable across the different treatments and conditions, and results are presented as the ratio protein of interest/eEF2.

### Statistics

A repeated-measures ANOVA, with as within-factor overload and between-factor age, was used to test for effects of overload and age on fatigue resistance. To test for the effects of (1) age and overload, or (2) resveratrol and overload on mean FCSA, mean (integrated) SDH, COX-4 and FLK-1 expression, CD, C/F, Log_rSD_ data, we used a univariate three-way ANOVA (IBM SPSS version 20) with the following factors: (1) age, muscle region (where appropriate) and overload, or (2) resveratrol, muscle region (where appropriate) and overload. In cases of fiber type-specific parameters (integrated) SDH, LCFR and CFD, the independent factor “muscle fiber type” was added to determine differences between fiber types. Effects were considered significant at *P* < 0.05. Data are expressed as means ± standard error of the mean (SEM).

## Results

### Effects of age, overload and resveratrol on fatigue resistance and potentiation

Table 1 shows that overload resulted in an increase in muscle mass and maximal tetanic force in both adult and old mice, irrespective of supplementation with resveratrol (*P* < 0.05) (Ballak et al. 2014a).

**Table 1:** Muscle mass, maximal isometric force, potentiation and fatigue resistance in control and overloaded *m. plantaris* of adult, old and old-res mice

	Adult mice (*n* = 7)		Old mice (*n* = 10)		Old-res mice (*n* = 10)	
	Con vs. Ovl		Con vs. Ovl		Con vs. Ovl	
Muscle mass (mg)	24.2 ± 0.7	32.1 ± 1.2*	22.4 ± 0.7**	28.1 ± 1.1*	23.8 ± 0.8	29.8 ± 0.9*
Max force (mN)	497 ± 34	663 ± 50*	470 ± 22	579 ± 36*	529 ± 30	557 ± 39*
Potentiation (%)	31.5 ± 5.6	29.2 ± 6.8	47.8 ± 6.8	35.4 ± 3.9	51.3 ± 4.3	19.3 ± 6.1*^,^***
FI_120/1_	49.9 ± 1.0	66.2 ± 4.9*	51.6 ± 8.2	70.0 ± 5.6*	58.0 ± 3.8	64.3 ± 5.2*
FI_120/max_	38.3 ± 1.4	51.8 ± 4.4*	34.2 ± 5.1	52.5 ± 5.5*	38.4 ± 2.5	54.3 ± 4.6*

Muscle mass was reported previously (Ballak et al. 2015). Max force: the maximal achieved force during the fatigue protocol. FI_120/1_: fatigue index for ~40 % of tetanic force (not reported here), dividing the force of the 120th contraction by the first. FI_120/max_: fatigue index for ~40 % of tetanic force (not reported here), dividing the force of the 120th contraction by the maximal achieved force during the fatigue protocol. Values are mean ± SEM

*Different from control condition at *P* < 0.05, **different from adult at *P* < 0.05, ***overload × resveratrol interaction at *P* < 0.05

**Fig. 1:**
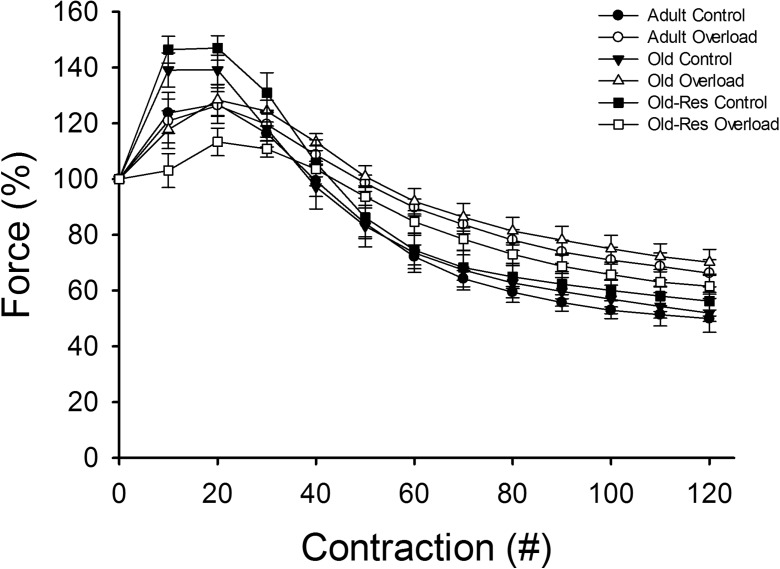
Effects of age, overload and resveratrol on in situ plantaris muscle fatigue resistance. Plantaris force (% of the force during the first contraction) is expressed as contraction number percentage of first contraction in the *m. plantaris* for adult (*circle*), old (*triangle*) and old-res (*square*) mice in both control (*black*) and overloaded (*white*) conditions

Figure 1 shows tetanic force normalized for the tetanic force of the first contraction during the fatigue test. The potentiation during the fatigue protocol was similar in adult and old muscles. Overload did not significantly alter potentiation in adult or old muscle. Resveratrol reduced potentiation in overloaded muscle only (resveratrol × overload interaction, *P* < 0.05; Table 1).

After potentiation, the force of the adult and old control muscles dropped rapidly to ~50 % of the initial value at the end of the test. Overload did result in an increased fatigue resistance; the forces at the end of the protocol were 65–70 % of the initial values (Table 1; Fig. 1; *P* < 0.05). Resveratrol did not significantly affect fatigue resistance.

### Effects of age, overload and resveratrol on muscle fiber size and muscle fiber type composition

In all groups, the FCSA of type IIB and IIX fibers was larger than that of type IIA fibers, and in the oxidative region of the *m. plantaris*, the FCSA of all fibers, except IIB fibers, was larger than those in the glycolytic part (Table 2; *P* < 0.01).

**Table 2:** Effects of age, overload and resveratrol supplementation on muscle fiber type area and fiber cross-sectional area

		FCSA (μm^2^)				Fiber type area %			
		ConG	ConO	OvlG	OvlO	ConG	ConO	OvlG	OvlO
Adult	IIA	458 ± 59	642 ± 37^e^	894 ± 123^b^	1046 ± 42^b,e^	7.1 ± 3.4	21.0 ± 1.4^e^	9.3 ± 3.0	30.9 ± 3.3^e^
	IIAX	503 ± 64	839 ± 83^e^	1066 ± 188^b^	1260 ± 49^b,e^	3.0 ± 3.2	5.6 ± 2.0	11.3 ± 1.7^b^	17.1 ± 1.9^b^
	IIX	1023 ± 123	1284 ± 65^e^	1616 ± 196^b^	1883 ± 125^b,e^	19.0 ± 7.4	29.8 ± 2.7	38.4 ± 5.8^b^	36.4 ± 4.0
	IIXB	1234 ± 400	1284 ± 334	1770 ± 172^b^	1875 ± 72^b,e^	0.9 ± 1.2	2.3 ± 1.5	9.4 ± 3.9^b^	2.6 ± 0.9
	IIB	1790 ± 185	1785 ± 71	2361 ± 221^b^	1868 ± 173^b^	70.0 ± 15.2	41.3 ± 2.7^e^	31.7 ± 9.2^b^	9.7 ± 3.5^e,b^
Old	IIA	504 ± 49	840 ± 70^e^	654 ± 98^a^	918 ± 146^e^	11.2 ± 2.2	34.1 ± 5.6	7.0 ± 1.7	34.5 ± 6.5
	IIAX	535 ± 86	1064 ± 193^e^	592 ± 83^a^	985 ± 106^e^	1.8 ± 0.1	2.9 ± 1.2^a^	2.7 ± 1.2^a^	7.5 ± 2.8^b,a^
	IIX	864 ± 80^a^	1408 ± 179^e^	1086 ± 102^a^	1355 ± 206^e,a^	25.8 ± 1.0	36.0 ± 3.2	27.2 ± 3.7^b^	34.1 ± 3.4
	IIXB	843 ± 174^a^	1398 ± 379^e^	1168 ± 186^b,a^	1543 ± 107^b,e^	1.6 ± 0.5	2.5 ± 0.8	3.0 ± 1.0^b,a^	4.6 ± 2.0
	IIB	1446 ± 201^a^	1929 ± 263	1726 ± 130^a^	1676 ± 144	60.4 ± 1.7^a^	24.6 ± 6.2	60.1 ± 6.0^b,a^	20.2 ± 5.8
Resveratrol	IIA	509 ± 62	757 ± 86^e^	873 ± 126^b,c^	986 ± 117^e,b^	4.3 ± 2.4^c^	24.1 ± 3.2^c,e^	6.0 ± 1.6	36.8 ± 3.6^e^
	IIAX	772 ± 92^c^	831 ± 129^e^	986 ± 132^c^	1116 ± 202^e^	1.6 ± 0.7	3.5 ± 0.9	5.7 ± 1.4^b^	9.9 ± 3.0^b^
	IIX	1270 ± 112^c^	1557 ± 153^e^	1608 ± 80^b,c^	1686 ± 134^e^	19.8 ± 2.7	34.1 ± 4.6	34.1 ± 5.0	31.9 ± 1.8
	IIXB	1547 ± 249^c^	1498 ± 288	1469 ± 84^c^	1227 ± 253	2.9 ± 1.1^c^	3.5 ± 1.7	10.8 ± 4.9^b,c^	3.6 ± 1.9
	IIB	1776 ± 137^c^	1970 ± 152	2115 ± 188^c^	1866 ± 183	71.4 ± 5.4^c^	34.9 ± 6.0^e^	43.5 ± 11.5	16.4 ± 4.5^e^

Glycolytic (ConG) and oxidative (ConO) regions of the *M. plantaris* in control condition were separated for analysis of FCSA and fiber type area %. The same was done for glycolytic (OvlG) and oxidative (OvlO) regions in overloaded condition. Values are mean ± SEM. Note that in muscle cross sections, low numbers of hybrid fibers were found, particularly type IIXB mounting up to about only 2–3 % of all fibers

^a^Significantly different compared to adult muscle (*P* < 0.05)

^b^Significantly different compared to control muscle (*P* < 0.05)

^c^Significantly different from old muscle (*P* < 0.05)

^d^Significantly different from glycolytic region

#### Age and overload

**Fig. 2:**
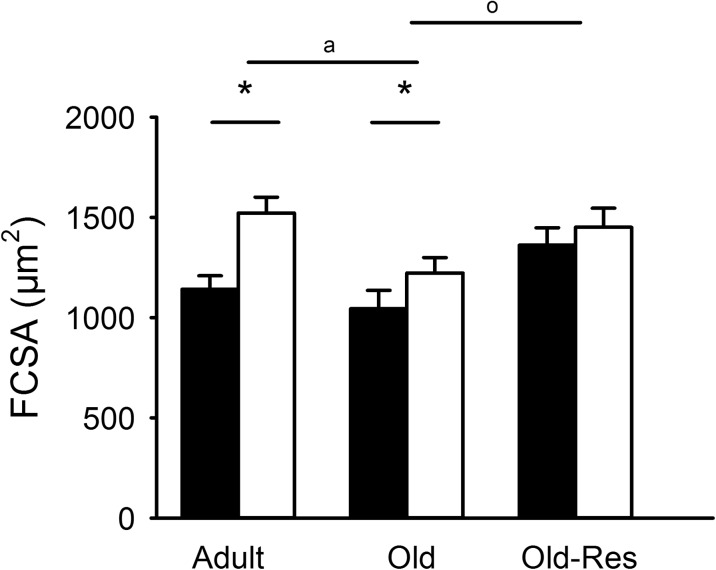
Effects of age, overload and resveratrol on *m. plantaris* FCSA. FCSA was smaller in old compared to adult muscle (^a^ *P* < 0.05), bigger in old-res than old muscle (^o^ *P* < 0.05) and increased after overload in adult and old (*P < 0.01), but not in old-res muscle. Values are represented as percentage of the adult control muscle; mean ± SEM

The FCSA was lower in old than adult muscle, but increased by overload in both groups (Fig. 2; *P* < 0.05). More specifically, overload increased the FCSA of type IIA, IIAX, and IIX fibers (*P* < 0.01), but more so in adult than old muscle (age × overload, *P* < 0.01; Table 2), indicating an attenuated hypertrophic response in old age.

#### Age and resveratrol

Resveratrol increased the pooled FCSA in both old control and old overloaded muscles (Fig. 2; *P* < 0.05) and specifically so in type IIAX, IIX, IIXB and IIB fibers (Table 2; *P* < 0.05). There was no significant resveratrol × overload interaction, indicating that resveratrol did not rescue the attenuated hypertrophic response.

#### Age and overload

Age did not significantly affect the area percentage of each fiber type. In adult, but not in old mice, overload did result in an increased proportion of the muscle occupied by type IIA, IIAX, IIX and IIXB fibers at the expense of type IIB fibers (age × overload, *P* < 0.05; Table 2).

#### Age and resveratrol

Resveratrol supplementation resulted in a 10 % larger areal percentage of IIB fibers (*P* < 0.05), which was obliterated after overload (resveratrol × overload, *P* < 0.05).

### Effects of age, overload and resveratrol on SDH activity

#### Mass-specific SDH activity is reflected by the SDH-OD

**Fig. 3:**
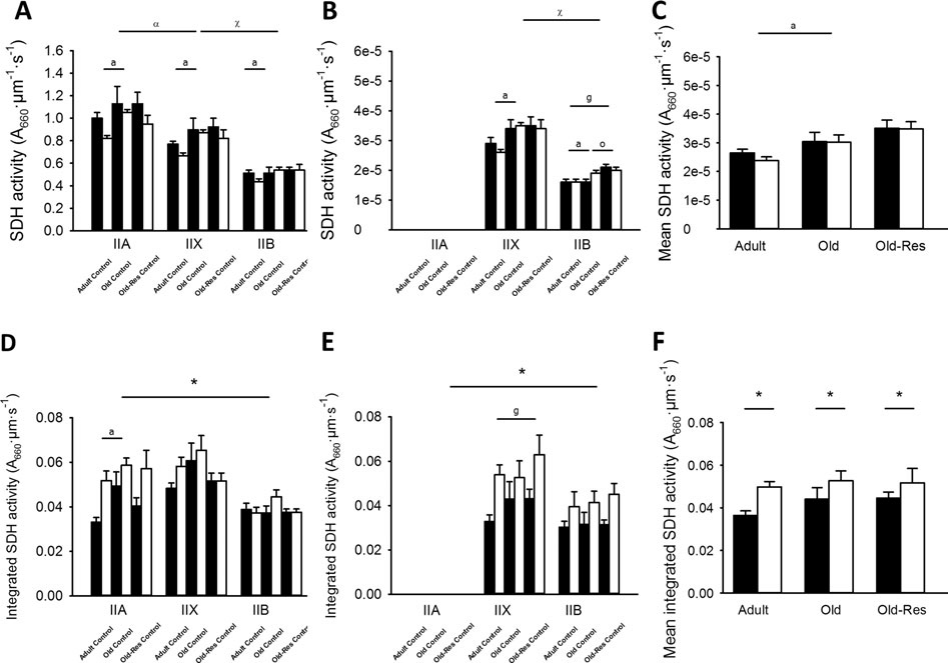
SDH activity and integrated SDH activity per fiber type for adult, old and old-res muscle. SDH activity for adult, old and old-res muscle in control and overloaded condition for type A, X, and B fibers in the oxidative (**a**) and glycolytic (**b**) region of the muscle. **c** Mean SDH for adult, old and old-res mice in both control (*black*) and overloaded (*white*) conditions. SDH activity in old muscle was higher than in adult muscle (^a^ *P* < 0.05). SDH activity was higher in old compared to adult muscle for all three fiber types (*P* < 0.05). Furthermore, the SDH activity in IIB of the glycolytic region of the muscle was lower than of type IIB fibers in the oxidative region (^g^ *P* < 0.05). Resveratrol did only increase SDH activity in type IIB fibers of the glycolytic region of the muscle (*P* < 0.05). SDH activity was lower in type IIB compared to IIX fibers (^χ^ *P* < 0.01) and lower in IIX compared to IIA fibers (^α^ *P* < 0.01). Integrated SDH activity for adult, old, and old-res muscle in control and overloaded condition for type A, X, and B fibers in the oxidative region (**d**) and glycolytic region (**e**) of the muscle. **f** Mean integrated SDH for adult, old, and old-res mice in both control (*black*) and overloaded (*white*) conditions. Integrated SDH activity increased after overload for all three fiber types (*P* < 0.01). Old muscle showed a higher integrated SDH activity in type IIA fibers, compared to adult (*P* < 0.01). Type IIX fibers had a lower integrated SDH activity in the glycolytic region, compared to the oxidative region (*P* < 0.01). Data shown are mean ± SEM

Type IIB fibers had the lowest and type IIA the highest SDH activity, with that of type IIX fibers in between (Fig. 3a, b; *P* < 0.01). Furthermore, SDH activity in IIB fibers in the glycolytic region was 10 % lower than that in the oxidative region of the muscle irrespective of age and resveratrol supplementation (Fig. 3a, b; *P* < 0.05).

#### Age and overload

The SDH activity was 20 % higher in old than in adult muscle (Fig. 3c; *P* < 0.05), suggesting a higher oxidative capacity in aged muscle. Overload did not significantly affect the SDH activity (*P* > 0.05).

#### Age and resveratrol

Resveratrol increased only the SDH activity of type IIB fibers in the glycolytic region by 28 % (Fig. 3b; *P* < 0.05).

#### Total SDH activity is reflected by the integrated SDH activity

Type IIB fibers had a 31 % lower integrated SDH activity than IIX fibers (*P* < 0.01), while the integrated SDH activity of IIA fibers was comparable to that of IIX fibers (*P* > 0.05). Type IIX fibers in the glycolytic region had a 14 % lower integrated SDH activity than those in the oxidative region of the muscle (*P* < 0.01).

#### Age and overload

The integrated SDH activity of type IIA fibers was 21 % lower in adult than old muscles (Fig. 3d, e; *P* < 0.01). Overload increased the integrated SDH irrespective of age, resveratrol supplementation, or fiber type (*P* < 0.01). The increase in integrated SDH activity, but not in SDH activity, suggests that mitochondrial biogenesis and fiber hypertrophy during overload were proportional.

#### Age and resveratrol

Resveratrol did not significantly affect the integrated SDH activity (Fig. 3d, e).

#### Mitochondrial protein expression

**Fig. 4:**
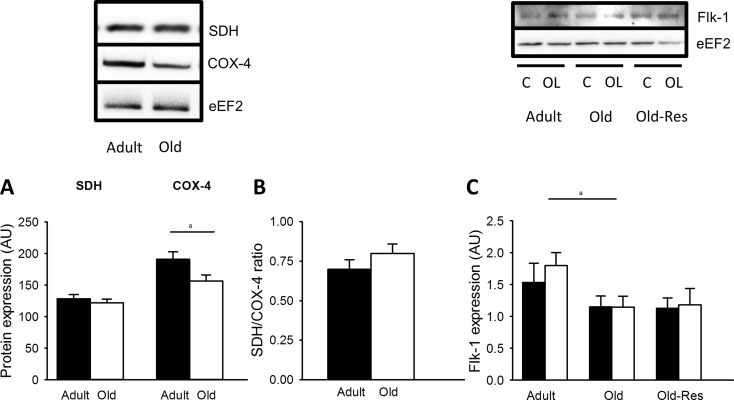
Flk-1, SDH, and COX-4 protein expression in adult, old and old-res muscle. **a** SDH protein expression was similar in adult and old muscle, while COX-4 protein expression was decreased in old muscle (^a^ *P* < 0.05). The SDH/COX-4 ratio (**b**) was not significantly different in aged muscle. **c** Flk-1 protein expression in *m. plantaris*, an important receptor for VEGF, was decreased in old compared to adult muscle (^a^ *P* < 0.05), but not affected by overload or resveratrol. The results are presented as the ratio protein of interest/loading control (eEF2). Values are mean ± SEM

In contrast to the elevated SDH activity, western blot analyses revealed that SDH protein expression was not significantly affected by age (Fig. 4a). This suggests that the specific activity of the SDH complex was increased by age. COX-4 protein expression did decrease with age (Fig. 4a; *P* < 0.05). Despite the lower COX-4 expression, the SDH/COX-4 ratio was not significantly altered by age (Fig. 4b; *P* > 0.05).

### Overall muscle capillarization

**Fig. 5:**
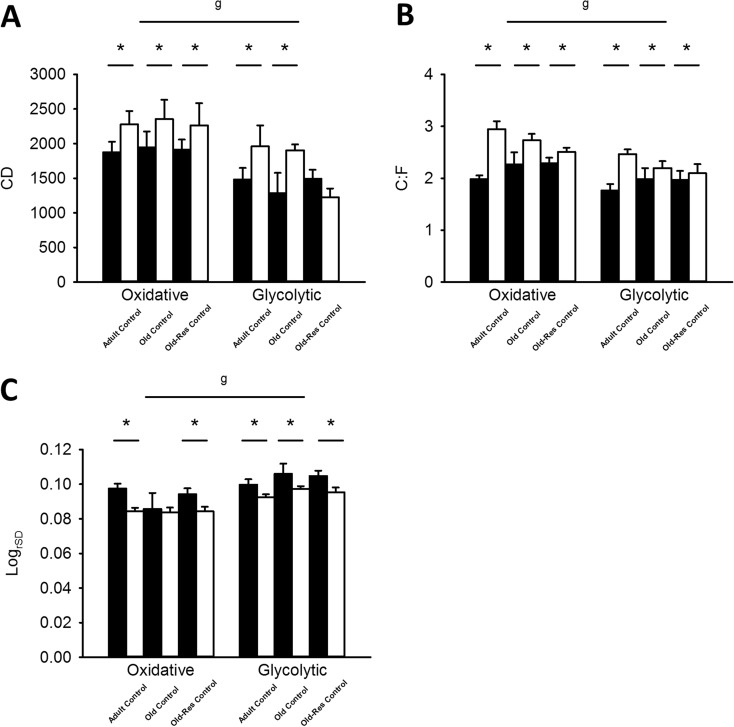
Effects of age, overload, and resveratrol on CD, C/F, and Log_rSD_. **a** CD was unaffected by age, however increased by overload (*P* < 0.01). Resveratrol blunted the overload-induced increase in CD, but only in the glycolytic region of the muscle (resveratrol × overload, *P* < 0.05). **b** C/F was increased by overload (*P* < 0.01), but unaffected by age or resveratrol. Both CD and C/F were higher in the oxidative region of the *m. plantaris* compared to the glycolytic region. **c** Log_rSD_ for adult, old and old-res control (*black*) and overloaded (*white*) muscle in the oxidative and glycolytic muscle regions. Log_rSD_ was higher in the glycolytic than in the oxidative region of the *m. plantaris* muscle (^g^ *P* < 0.01), but not affected by age. Overload reduced Log_rSD_ (*P < 0.01), while resveratrol did not affect the Log_rSD_ (*P* > 0.05). Values are mean ± SEM. *Black bars* control situation; *white bars* overload situation

Both CD and C/F were significantly higher (~35 and ~18 %, respectively) in the oxidative than the glycolytic region of the muscle (*P* < 0.01; Fig. 5a, b). Log_rSD_ was 11 % higher in the glycolytic than in the oxidative region of the *m. plantaris* muscle (*P* < 0.01), indicating a larger heterogeneity in capillary spacing in the glycolytic than in the oxidative region of the muscle.

#### Age and overload

**Fig. 6:**
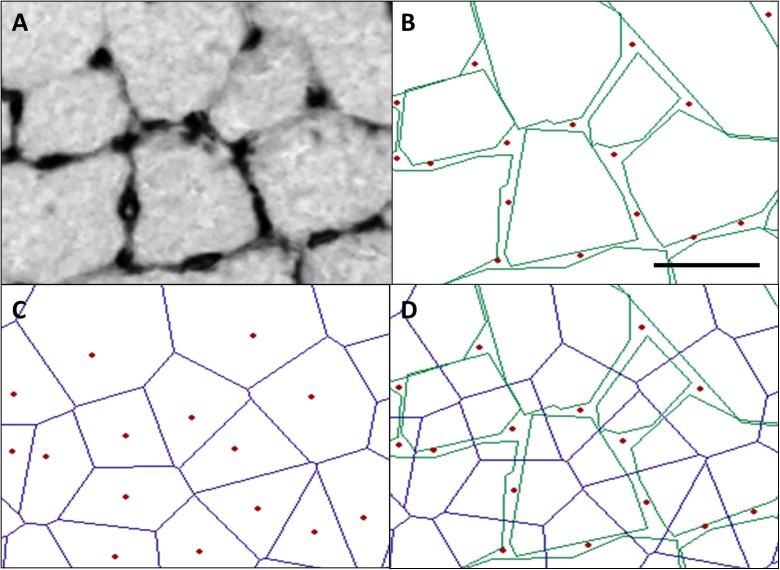
Illustration of the analyses of capillary data. **a** Typical example of capillaries identified with lectin staining in mice muscle. **b** In *red*, the capillaries, and in *green*, the muscle fiber outlines are shown, while in *blue* (**c**), the calculated capillary domains are depicted. **d** The overlap of domains and fibers, used to calculate fiber-specific capillary supply, as described in the methods section. *Bar* represents 30 μm (color figure online)

CD was not significantly affected by age, but increased ~20 % by overload (*P* < 0.01). C/F was unaffected by age, but increased by overload (*P* < 0.01; Fig. 5b) and more so in adult (44 %) than old (16 %) muscle (age × overload, *P* < 0.05). Log_rSD_ was not significantly affected by age (Fig. 5), but overload reduced Log_rSD_ by 9 %, indicating a more homogeneous distribution of the capillaries in the hypertrophied muscles (*P* < 0.01). Figure 6 illustrates the analysis of capillary data.

#### Age and resveratrol

In resveratrol-treated muscle, overload only increased CD in the oxidative region by 18 % (resveratrol × overload, *P* < 0.05; Fig. 5a). Resveratrol, however, did not significantly affect C/F or Log_rSD_ (*P* > 0.05).

### Muscle fiber-specific capillarization

**Fig. 7:**
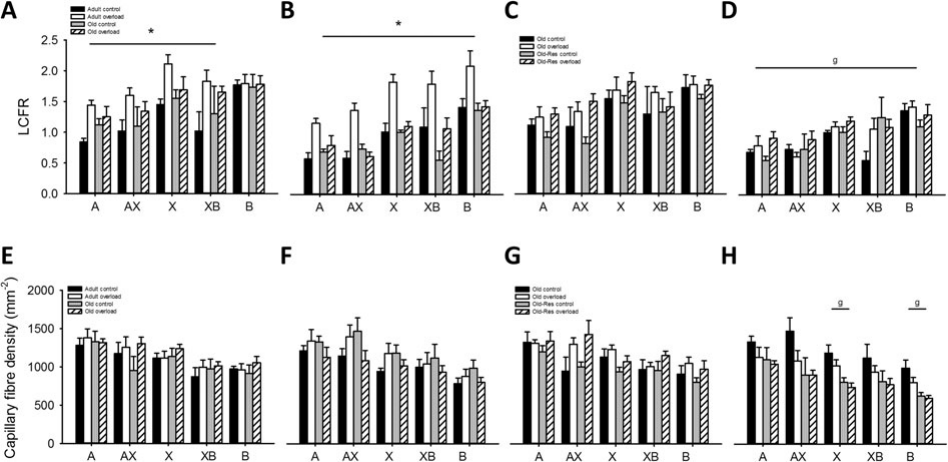
Effects of age, overload, muscle area and resveratrol supplementation on local capillary to fiber ratio and capillary fiber density. LCFR for adult control (*black*), overloaded (*white*), old control (*dashed*), and overloaded (*striped*) muscle for type A, X, and B fibers in the oxidative (**a**) and glycolytic (**b**) region of the muscle. LCFR activity for old control (*black*), overloaded (*white*), old-res control (*dashed*) and overloaded (*striped*) muscle for type A, X, and B fibers in the oxidative (**c**) and glycolytic (**d**) region of the muscle. Overload increased the LCFR in fibers of all types in both regions (*P* < 0.05), except for oxidative type IIB fibers (*P* > 0.05). An interaction effect (age × overload) showed that the increase in LFCR was larger for adult than for old muscle (*P* < 0.05). Resveratrol did not affect the LCFR (*P* > 0.05). For both old and old-res muscle, the LCFR was higher in the oxidative, compared to the glycolytic part (*P* < 0.01). CFD (mm^−2^) for adult control (*black*), overloaded (*white*), old control (*dashed*), and overloaded (*striped*) muscle for type A, X, and B fibers in the oxidative (**e**) and glycolytic (**f**) region of the muscle. CFD (mm^−2^) for old control (*black*), overloaded (*white*), old-res control (*dashed*) and overloaded (*striped*) muscle for type A, X and B fibers in the oxidative (**g**) and glycolytic (**h**) region of the muscle. CFD showed an age × overload interaction for type IIAX and IIX fibers, only in the glycolytic region (*P* < 0.05). Resveratrol decreased the CFD for IIAX, X and B fibers in the glycolytic region of the muscle (*P* < 0.05). For type AX fibers, overload increased the CFD in both old and old-res muscle (*P* < 0.05). Furthermore, in IIX and IIB fibers CFD was lower in the glycolytic, compared to the oxidative region (age × overload interaction; *P* < 0.05). Data shown are mean ± SEM

For all groups, LCFR was higher in the oxidative than in the glycolytic region (Fig. 7; *P* < 0.01).

#### Age and overload

LCFR of all muscle fiber types was not significantly affected by age. However, overload increased LCFR in fibers of all types (Fig. 7a, b; *P* < 0.05), except that of type IIB fibers in the oxidative region (Fig. 7a). The overload-induced increase in LCFR was 57 % in adult versus 14 % in old muscle (age × overload, *P* < 0.05), indicating attenuated angiogenesis in old overloaded muscles. In most cases, the CFD was not significantly affected by age or overload (Fig. 7e, f), but there was a significant age × overload interaction (*P* < 0.05) for type IIAX and IIX fibers, reflected by a decrease in CFD in old, but not adult, overloaded plantaris muscle.

#### Age and resveratrol

Resveratrol did not significantly affect LCFR (Fig. 7c, d). In both old and old-res, CFD of type IIX and IIB fibers was lower in the glycolytic than the oxidative region of the muscle (Fig. 7g–h; age × overload interaction, *P* < 0.05). Resveratrol decreased CFD of IIAX fibers in the glycolytic region of the muscle by 26 % (resveratrol × region interaction, *P* < 0.05).

### VEGF and Flk-1 protein expression

Flk-1 protein expression, a receptor for VEGF, was 31 % lower in the *m. plantaris* from old than young-adult mice (Fig. 4c; *P* < 0.05), irrespective of overload or resveratrol supplementation.

### Overload affects inverse relationship between SDH and FCSA

**Fig. 8:**
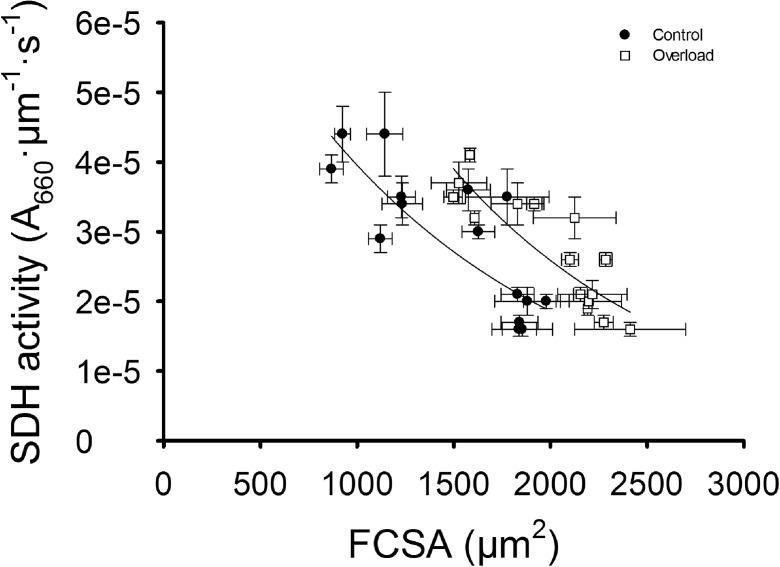
SDH activity and FCSA are inversely related. The figure shows the relationship between mean SDH activity per muscle and FCSA for adult and old muscles. Overload affects this relationship by inducing a rightward shift (*R* ^2^ = 0.74; *P* < 0.01), compared to the control (*R* ^2^ = 0.67; *P* < 0.01). The *black lines* represent the best fit hyperbola. Values are mean ± SEM

Figure 8 shows that in the muscles from both adult and old mice, there is an inverse relationship between SDH activity and FCSA (*R*^2^ = 0.74 and *R*^2^ = 0.67, respectively, both *P* < 0.01). Overload induced a rightward shift of the fitted hyperbola.

The LCFR of adult and old muscle correlated strongly with the FCSA (*R*^2^ = 0.52, *P* < 0.05), but not with the SDH activity (*R*^2^ = 0.07, *P* > 0.05), suggesting that the capillary supply to a fiber is more determined by fiber size than fiber oxidative capacity.

## Discussion

Here, we investigated whether sarcopenia and the blunted hypertrophic response in old mouse muscle (1) are partly due to a reduced capillarization and angiogenesis, that are (2) accompanied with a reduced oxidative capacity and fatigue resistance in old control and overloaded muscles, and that (3) can be rescued by the anti-inflammatory antioxidant resveratrol. The main observations were that both overload-induced hypertrophy and angiogenesis of the *m. plantaris* were attenuated in old mice. Despite the diminished angiogenic response and the unaltered aerobic capacity, the fatigue resistance was increased to a similar extent in adult and old *m. plantaris* of C57BL/6J mice after 6 weeks overload. The proportional increase in aerobic capacity and muscle fiber size after overload was unexpected as the inverse relationship between fiber size and oxidative capacity (van der Laarse et al. 1998) suggests that oxygen diffusion limitations may impose a size constraint on muscle fibers. The more homogeneous distribution of capillaries in the overloaded muscles improves muscle oxygenation (Degens et al. 1994a; Goldman et al. 2006; Turek et al. 1989) and may make such an adaptation possible in vivo. While resveratrol supplementation did rescue some of the age-related atrophy, it did not affect the fatigue resistance or rescue the angiogenic and hypertrophic response in old age. It is thus possible that impaired angiogenesis contributes to the attenuated hypertrophic response in old age, which cannot be rescued by resveratrol.

### Age-related changes in muscle capillarization and oxidative capacity

An adequate capillarization is vital for muscle function, and any impairment in oxygen delivery could have major implications for fatigue resistance. It has been observed in Wistar rats (Degens et al. 1993b) and humans (Proctor et al. 1995) that the capillary supply to fibers is reduced in old age, suggesting an age-related capillary rarefaction. In the present study, both the capillary-to-fiber ratio and the capillary density were similar in adult and old mouse *m. plantaris*, which suggests that in 25-month-old mice, the capillary bed had not yet been significantly affected by age. Nevertheless, we did find a reduced Flk-1 expression in old muscle, similar to a previous study in mice (Wagatsuma 2006), indicative of a reduction in angiogenic signaling in old muscle and possibly an early indication of problems to maintain the vascular bed.

SDH, or complex II, is both part of the citric acid cycle and the respiratory chain. While it has been reported that the SDH activity in the muscle decreases with age (Proctor et al. 1995; Sczelecki et al. 2014), we and others (Doran et al. 2008) found an increased SDH activity in old compared to adult *m. plantaris*. In western blots, however, we observed no age-related change in the expression of SDH, suggesting that the specific activity of the SDH complex was increased. The age-related increase in SDH activity in our study may be a compensation for the decrease in COX expression, which has also been observed by others (Bua et al. 2006; Lee et al. 1998), in an attempt to maintain aerobic capacity.

The absence of any age-related differences in fatigue resistance during a series of intermittent isometric contractions in our study was similar to that observed in the *m. plantaris* of 13- and 25-month-old Wistar rats (Degens et al. 1993a). This observation was unexpected, considering the age-related increase in the SDH activity and the type IIB to type IIA fiber type transition. Similarly, after overload, the fatigue resistance was increased without a change in SDH activity. The dissociation of changes in fatigue resistance and oxidative capacity suggest that the fatigue test, while useful to distinguish fatigable and fatigue resistant motor units (Larsson et al. 1991) and to predict the aerobic capacity in single muscle fibers, is not as powerful a predictor of aerobic capacity in whole muscle (Degens and Veerkamp 1994). The reason for this may be related to the more severe increase in metabolites during whole muscle fatigue than during single motor unit fatigue development (Gardiner and Olha 1987). If indeed lactate accumulation from glycolytic fibers has a negative impact on the neighboring fatigue-resistant aerobic fibers, one may expect that a higher capillarization will enhance the removal of lactate and be associated with a higher fatigue resistance. Consistent with this, we found that changes in fatigue resistance during aging and overload paralleled changes in muscle capillarization. Nevertheless, in rats older than 26 months, there was a significant reduction in fatigue resistance with a similar test (Degens and Alway 2003). This and our previous observations that 25-month-old mice already show age-related deteriorations in determinants of muscle force generating capacity similar to 60–70-year-old humans (Ballak et al. 2014b) is in line with our suggestion that these mice present a model for early age-related changes in muscle structure and function.

### Effects of mechanical overload in old age on muscle morphology and fatigue resistance

Overload increased the FCSA more in adult than in old muscle, confirming the blunted hypertrophic response in old age (Ballak et al. 2015; Degens and Alway 2003). In both adult and old muscle, hypertrophy was accompanied by a proportional increase in the number of capillaries per fiber and fiber size as reflected by the maintained capillary fiber density. However, the increase in LCFR, reflecting the number of capillaries per fiber, was less in old than in adult hypertrophied muscles, indicating impaired angiogenesis. Part of the impaired angiogenic response may be related to the age-related decrease in Flk-1 protein expression, the receptor for VEGF, an important endothelial mitogenic factor (Wagatsuma 2006). An impaired angiogenic response has also been seen in ischemic hind limbs that was due to impaired VEGF expression and HIF-1α activity in muscles of old animals (Rivard et al. 2000; Rivard et al. 1999). We and others have found a positive relationship between the size of a fiber and the capillary supply in both normal (Ahmed et al. 1997; Wust et al. 2009) and hypertrophied muscle (Degens et al. 1994b) and a similar time course of hypertrophy and angiogenesis (Egginton et al. 2011; Plyley et al. 1998). Whatever the cause, the impaired angiogenesis we observed in the old muscle may thus well contribute, in addition to a reduced Id2 expression and lower satellite cell number (Ballak et al. 2015), to the blunted hypertrophic response in old age.

The elevated fatigue resistance despite unaltered aerobic capacity in overloaded muscles may be attributable to an increased proportion of slower fibers (type IIB to IIA shift), that are more economical than faster fibers during isometric contractions (Stienen et al. 1996). However, we observed a greater shift to slower fibers in adult than old muscle, while the overload-induced increase in fatigue resistance was similar in adult and old muscle. In theory, an enhanced myoglobin concentration could facilitate the diffusion of oxygen (Kreuzer and Hoofd 1987), but this has been shown to not significantly change during overload (Masuda et al. 1997). Interestingly, the CD was increased and the distribution of capillaries became more homogeneous in both adult and old overloaded muscle, which is expected to improve the oxygenation of the muscle (Degens et al. 2006; Turek et al. 1991) and thereby potentially contribute to the enhanced fatigue resistance in the overloaded muscles. Further support for the potential significance of the distribution of capillaries in muscle fatigue resistance is the absence of a change in both CD and heterogeneity of capillary spacing on the one hand and fatigue resistance on the other during aging. It is admittedly a tenuous link that needs further investigation.

### Effect of resveratrol on muscle morphology and muscle fatigue in old overloaded muscle

Resveratrol is thought to induce its effects via Sirt-1 (Yun et al. 2012) and/or AMP-activated protein kinase activation (Park et al. 2012) that controls PGC-1α expression (Lagouge et al. 2006). PGC-1α plays an important role in mitochondrial biogenesis (Goffart and Wiesner 2003) and has been reported to also stimulate angiogenesis (Olesen et al. 2010). Correspondingly, resveratrol supplementation has been shown to improve mitochondrial function, endurance performance and muscle fatigue resistance in rodents (Hart et al. 2013; Lagouge et al. 2006; Murase et al. 2009; Selsby et al. 2012). In contrast to our expectation, resveratrol did not improve capillarization, mitochondrial function, or fatigue resistance. A recent study also did not find any beneficial effects of resveratrol on muscle capillarization in 15-month-old mice (Ringholm et al. 2013) and it has even been shown to limit training-induced angiogenesis in old men (Gliemann et al. 2014). The discrepancy in the published literature and our findings may be related to the dose of resveratrol and the degree of oxidative stress in the muscle (Bosutti and Degens 2015), where we administered 0.4 % resveratrol per gram body mass and others a lower dose (0.05 % per gram body mass). It is also possible that the beneficial effects of resveratrol on fatigue resistance and the capillary bed only become apparent when muscle capillarization is already impaired.

### Relationship between fiber size and aerobic capacity

The inverse relationship between SDH activity and FCSA was suggested to be determined by diffusion limitations (Degens 2012). Such an inverse relationship was also observed in our study and did not change during aging. To our surprise, however, the relationship in overloaded muscles was shifted upward, indicating that the maximal oxygen uptake for a fiber with a given FCSA was larger in overloaded than control muscles. It is likely that the improved homogeneity of capillary distribution that improves muscle oxygenation (Degens et al. 1994a; Turek et al. 1991) has made this adaptation possible.

## Conclusion

In conclusion, even though capillarization in muscles from old and adult mice was similar, overload-induced angiogenesis was blunted in old mice. The impaired angiogenic response may at least partly contribute to the blunted hypertrophic response in old age. Despite the attenuated angiogenesis, the fiber hypertrophy and mitochondrial biogenesis in response to overload were all proportional, so that the relationships between fiber capillarization, aerobic capacity, and size were similar in control and overloaded muscle of both adult and old mice.

The increased total oxidative capacity of a fiber of a given size and the increased fatigue resistance of overloaded muscles may be partly attributable to an increased capillary density and a more homogeneous distribution of capillaries. Resveratrol supplementation attenuated age-related muscle wasting, but did not improve fatigue resistance or capillarization.
